# Supplementary material for: Rare Bacteria Can Be Used as Ecological Indicators of Grassland Degradation
Source: Microorganisms. 2023 Mar 15;11(3):754. doi: 10.3390/microorganisms11030754 (PMC10058834; doi:10.3390/microorganisms11030754)

Supplementary materials

Rare bacteria can be used as ecological indicators of grassland degradation

Mengjun Liu [1]; Yue Ren [1]; Weihong Zhang [2,3,*]

[1] Institute of Animal Husbandry and Veterinary Science, Tibet Academy of Agricultural and Animal Husbandry Sciences, Lhasa, 850000, China

[2] Key Laboratory of Aquatic Botany and Watershed Ecology, Wuhan Botanical Garden, Chinese Academy of Sciences, Wuhan, 430074, China

[3] University of Chinese Academy of Sciences, Beijing, 100049, China

Table S1. Information on the vegetation coverage and soil physicochemical properties in the typical mountain meadow of Qinghai-Tibet Plateau.VC = vegetation coverage, SWC = soil water content, TN = total nitrogen, TOC = total organic carbon, TP = Total phosphorus, HN = hydrolyzable nitrogen, AP = available phosphorus.

Samples	VC (%)	pH	SWC (%)	TN (g/kg)	TOC (g/kg)	TP (g/kg)	HN (mg/kg)	AP (mg/kg)
GS1	5.25	7.16	7.96	1.10	13.18	0.43	70.39	4.50
GS2	11.38	7.21	11.52	1.29	13.85	0.35	107.58	6.88
GS3	7.92	7.19	13.68	1.15	13.55	0.41	89.23	5.74
GS4	11.15	6.85	10.80	0.91	8.20	0.24	71.00	5.40
GS5	19.12	7.07	11.44	0.92	9.41	0.23	90.14	5.81
GS6	16.67	6.93	32.75	1.24	16.25	0.27	122.00	8.60
GS7	69.96	7.87	15.50	2.61	34.56	0.43	205.55	15.53
GS8	48.68	7.16	16.99	2.71	32.40	0.44	198.00	16.90
GS9	41.85	7.20	17.71	2.74	41.33	0.47	196.20	18.03
GS10	30.54	7.27	18.93	2.10	24.19	0.32	151.52	8.49
GS11	63.45	7.02	21.08	2.83	39.68	0.53	218.34	13.91
GS12	43.76	7.04	19.54	2.57	32.06	0.49	213.43	8.53
GS13	21.97	7.51	0.13	1.92	23.35	0.38	126.33	12.18
GS14	19.26	7.40	0.12	1.73	23.22	0.34	108.02	12.18
GS15	21.62	7.31	0.12	1.85	24.84	0.43	121.40	13.17
GS16	31.20	7.03	0.12	2.35	30.64	0.49	180.10	25.27
GS17	46.70	7.26	0.13	2.09	26.90	0.35	171.00	12.30
GS18	18.02	7.37	0.13	1.62	23.05	0.50	111.78	9.63

Table S2. The relationship between the assembly processes of abundant and rare bacterial taxa in degraded grassland soils with their corresponding vegetation characteristics and soil physicochemical and nutrients. Asterisks denote levels of significance (* *p*-value < 0.05, ** *p*-value < 0.01).

	Whole	Abundant	Rare
Vegetation coverage	-0.051	-0.01	0.189*
pH	0.040	0.211**	0.194*
SWC	0.041	0.102	0.272**
TN	-0.159*	-0.136	0.304**
TOC	-0.116	-0.181*	0.297**
TP	-0.214**	-0.272**	0.179*
AN	-0.037	-0.193*	0.253**
AP	-0.041	0.162*	-0.001

Figure S1. Comparison of niche width between the abundant and rare bacterial taxa. Asterisks denote levels of significance (*** p-value < 0.001).

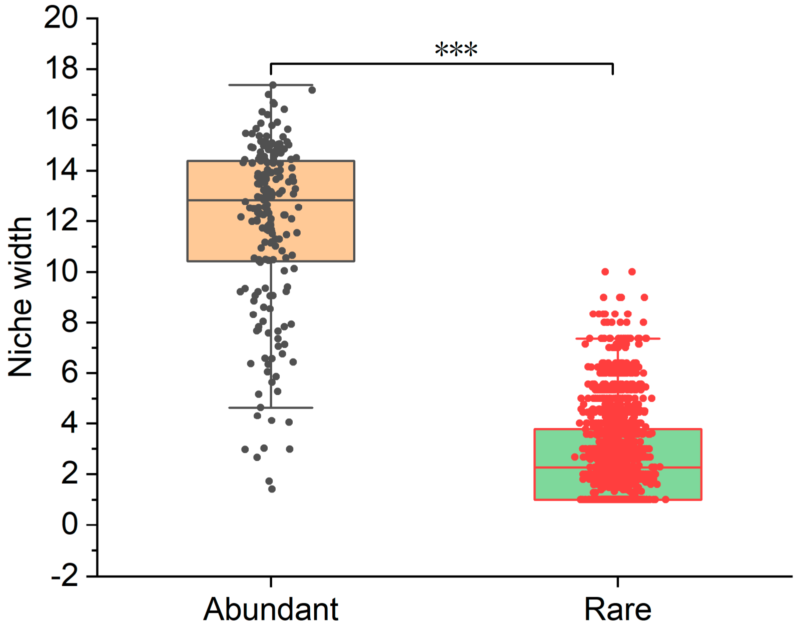

Figure S2. The distribution characteristics of the connectivity degree in the co-occurrence network, and the power function mean that was a scale-free small-world network.

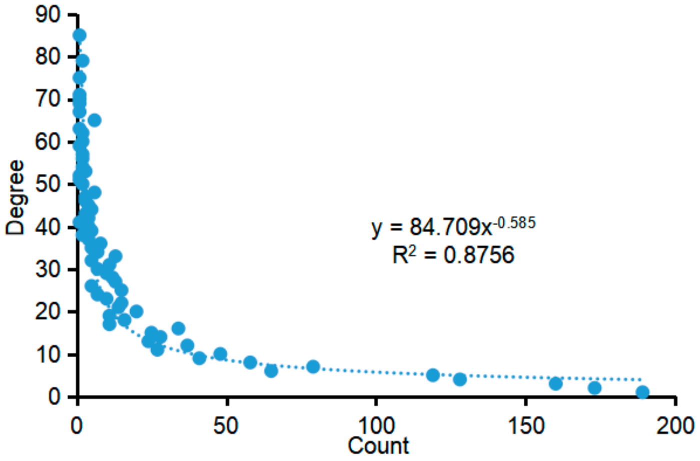

Supplement: Supplementary file 1 [file microorganisms-11-00754-s001.zip › microorganisms-2253643-SI.pdf]
